# Association between interpersonal relations and anxiety, depression symptoms, and suicidal ideation among middle school students

**DOI:** 10.3389/fpubh.2023.1053341

**Published:** 2023-02-14

**Authors:** Meixin Zheng, Xiaoyan Guo, Zhiyan Chen, Jing Deng, Mi Hu

**Affiliations:** ^1^Department of Social Medicine and Health Management, Xiangya School of Public Health, Central South University, Changsha, China; ^2^Department of Epidemiology and Health Statistics, Xiangya School of Public Health, Central South University, Changsha, China

**Keywords:** middle school students, interpersonal relations, anxiety symptoms, depressive symptoms, suicidal ideation

## Abstract

**Objective:**

This study aimed to explore the relationship between different types of interpersonal relationships and anxiety symptoms, depressive symptoms, and suicidal ideation and discusses the impact of different grades among middle school students.

**Methods:**

The Patient Health Questionnaire Depression Scale, the Chinese version of the Generalized Anxiety Scale, suicidal ideation questions, and interpersonal relations items were used to measure the depression symptoms, anxiety symptoms, suicidal ideation, and interpersonal relations of the participants. The variables of anxiety symptoms, depressive symptoms, suicidal ideation, and interpersonal relations were screened using the Chi-square test and principal component analysis. AMOS17.0 constructs the path of the association between interpersonal relations and depressive symptoms, anxiety symptoms, and suicidal ideation.

**Results:**

The mother-child relationship had direct impacts of −0.06, −0.07, and −0.06 on anxiety symptoms, depressive symptoms, and suicidal ideation. On anxiety symptoms, depressive symptoms, and suicidal ideation, the direct impacts of the father-child relationship were −0.09, −0.03, and −0.08. Moreover, the direct effects of peer relationships on depressive symptoms were −0.04, whereas the direct impact of teacher-student relationships on anxiety and depressive symptoms were −0.10 and −0.09. Further pathway analysis based on grade level showed that in the junior high school model, the direct effect of the mother-child relationship on anxiety and depressive symptoms was −0.18 and −0.16. The direct impact of the father-child relationship on depressive symptoms and suicidal ideation was −0.08 and 0.09. The direct effect of peer relationships on depressive symptoms was −0.08, and the direct impact of the teacher-student relationship on anxiety symptoms was −0.06. In the high school model, the direct effect of the mother-child relationship on suicidal ideation was −0.07, while the direct impact of the father-child relationship on anxiety, depression, and suicidal ideation was −0.10, −0.07, and −0.12, respectively. In addition, the direct effects of peer relationships on anxiety and depression were −0.06 and −0.05, and the direct impact of teacher-student relationships on anxiety and depression was −0.10 and −0.11.

**Conclusion:**

The father-child relationship affects suicidal ideation and depression the most, followed by the mother-child relationship, the teacher-student interaction, and the peer relationship. The teacher-student relationship influences anxiety symptoms the most, followed by the father-child and mother-child relationships. The association between interpersonal interactions and anxiety, depressive symptoms, and suicidal ideation varied significantly across grade levels.

## 1. Introduction

Depression and anxiety among high school students are essential public health issues. According to the 2019 Global Burden of Disease Study, depression and anxiety are the fourth and sixth leading causes of impairment in teenagers (1). In China, the prevalence of depression and anxiety among middle school students ranged from 10.9 to 20.4% and 11.0 to 23.5% from 2001 to 2021, respectively (2–7). Suicide is one of the world's biggest health problems (8) and the second leading cause of death among teenagers aged 15–19 (9). A meta-analysis of the prevalence of suicide-related behaviors among Chinese middle school students showed that 17.7% of middle school students have suicidal ideation (10).

In addition, the prevalence of depression and anxiety co-morbidity is significant. Seventy-two percent of patients with lifelong anxiety disorders had a history of depression, and 48% of patients with lifelong depression had anxiety disorders, according to cohort research (11). 53.3 % of Changsha middle school students with depressive symptoms also had anxiety symptoms (12). Students with depressive symptoms have a high risk of committing suicide (13). There is a high relationship between anxiety, depressive symptoms, and suicidal ideation; anxiety and depressive symptoms are independent influences on suicidal ideation (14, 15). Depression and anxiety symptoms can have a substantial impact on all parts of the life of middle school children. For example, depression and anxiety can significantly decrease middle school students' academic performance, educational level, educational attainment (16, 17), and physical fitness (18) and even threaten their lives in severe cases (19). Middle school students with depression and anxiety who do not receive timely and effective treatment are more likely to develop mental illness as adults (20–23).

Interpersonal relationships are defined by social psychology as the psychological distance and behavioral tendencies between individuals and others (24). Middle school pupils' most significant adolescent relationships are with their peers, teachers, and parents (25–27). Consequently, based on the types of interpersonal interactions among middle school students, the interpersonal relationships among middle school students can be categorized as parent-child, peer, and teacher-student. The literature shows that the influence of mother-child and father-child relationships on middle school students is different (28, 29). Therefore, our study divided the parent-child relationship into the mother-child relationship and the father-child relationship and divided the interpersonal relationships of this research into four dimensions: the mother-child relationship, the father-child relationship, the peer relationship, and the teacher-student relationship.

Depression and anxiety symptoms of middle school students are greatly influenced by their interpersonal relationships (30, 31). According to Attachment Theory (32), adolescents with positive interpersonal relationships are less likely to exhibit depression and anxiety symptoms (33). A longitudinal study showed that a warm and intimate parenting style could predict a future reduction in suicidal behavior (33). The parent-child relationship is closely related to the mental health of children and adolescents (34, 35). A longitudinal study indicates that warm and intimate parenting can predict a reduction in suicidal behavior in the future. Another longitudinal study revealed that the closer the relationship between adolescent mother and child, the lower the risk of early adulthood suicidal ideation (36). In a parent-child connection, parental caring is high, and teenage depressive symptoms are low (37). In clinical samples of adolescents with suicidal ideation, the adolescents' cognition of family support acts as a protection to reduce the association between anxiety and suicidal ideation (38). Numerous research demonstrates that suicidal ideation is linked to low-quality peer connections, lack of friends, low-level peer intimacy, high-level peer exclusion, and peer bullying (39, 40). The American Adolescent Health Cohort Study revealed in 2006 that the parent-child relationship was highly associated with the risk of suicide. However, there is no correlation between peer relationships, teacher-student relationships, and the risk of suicide (41). In contrast, the results of the related study are inconsistent. In 2018, Madjar et al. utilized the Hierarchical Linear Model to conclude that parent-child and peer relationships were significantly correlated with suicidal ideation at the individual level, whereas the teacher-student relationship was significantly associated with suicidal ideation (42).

Early studies in China found that the parent-child relationship plays a significant role in shaping adolescent mental health (43–45). Students from families with poor parent-child communication display more depressive symptoms (44). In a clinical sample of adolescents with suicidal ideation, we discovered that the cognition of family support among middle school students was a protective factor that weakened the connection between anxiety symptoms and suicidal ideation (45). Peer relationships are among the most crucial partnerships for teenagers (46). Zhou et al. found that interpersonal relations within the classroom and the classroom teacher's management philosophy influenced the development of depressive symptoms in secondary school students, with depressive symptoms being more prevalent in students with strict management, few friends, or discordant classroom relationships (47). These previous studies in China have focused more on the association between single Interpersonal relations and Depression and anxiety symptoms, lacking a comprehensive comparison of the effects of the roles of parents, peers, and teachers on the Depression and anxiety symptoms of middle school students.

In addition, an earlier study on the effect of interpersonal relationships on the mental health of middle school students paid limited attention to its developmental aspects. The impact of interpersonal relationships on middle school students' anxiety, depression, and suicidal ideation, as well as their patterns, may vary with age. According to the ecosystem theory, the family is the essential unit in the human growth behavior pattern. It has a significant and far-reaching effect on individuals' healthy development and social adaptability. Peer and teacher-student relationships will become increasingly crucial as microsystems develop (48). Wo et al. discovered that the peer relationship of middle school students was higher than the teacher-student and parent-child relationships. However, parent-child relationships improved during high school (49). Other research findings indicate that the parent-child relationship has a more significant impact on depression than other relationships, particularly in senior high school. According to the results of Liu et al., there are more peer conflicts in seventh grade, and parent-child conflicts increase the risk of depression in adolescents. In Grade 10, parental support was stronger than in Grade 7, whereas peer support was weaker (50). To investigate the impact of grade differences, this study will also examine the path and size of the correlation between interpersonal relationships and anxiety, depression, and suicidal ideation among middle school students at different stages of development (junior high school and senior high school).

In conclusion, poor interpersonal relationships significantly contribute to depressive and anxious symptoms. Interpersonal relationships are an intervenable factor, and they can be used as a breakthrough point to intervene in the depression and anxiety symptoms of teenagers (51, 52). Existing studies have confirmed the strong association between interpersonal relationships and depression and anxiety symptoms in middle school students. Still, they do not provide consistent information regarding the relative importance of parents, peers, and teachers during this developmental stage. Therefore, further studies are needed to determine the mechanisms underlying the influence of interpersonal relationships on the depressive and anxious symptoms of middle school students. This study aimed to construct the path and size of the association between interpersonal relations and depressive symptoms, anxiety symptoms, and suicidal ideation, as well as the impact of different grades.

## 2. Methods

### 2.1. Study participants and sampling procedure

The study employed a cluster sampling design with multiple stages. According to the classification standard of the Chinese Ministry of Education, middle schools in Changsha are categorized as junior high schools, senior high schools, vocational high schools, and junior high schools combined type. Then, according to the classification, four middle schools are selected. With the whole-group sampling method, 71 classes containing 3,669 Middle school students were selected using the class-based selection technique. The online survey method was adopted, and the teachers conducted and managed class-based group measurements. Before the survey was conducted, subjects and their parents were given opt-out informed consent. The IRB approved the Central South University Xiangya School of Public Health study.

### 2.2. Measurements

#### 2.2.1. Depressive symptoms

The Patient Health Questionnaire (PHQ-9) was used to evaluate the symptoms of depression in middle school students. The questionnaire was developed from the Diagnostic and Statistical Manual of Mental Disorders, Fourth Edition (DSM-IV), and contains nine items. The sum of the 9-item scores is the total questionnaire score, which ranges from 0 to 27, and each of the 9-item scores has four levels (0-3), which are used to assess intuitive feelings in the last 2 weeks, with higher scores indicating more severe depressive symptoms. A score of ≥10 is the cut-off value for a positive depressive symptoms screen (53). The scale has good reliability and validity in foreign studies (54, 55). The scale is also widely used in China and has good reliability and validity (56, 57). In this study, Cronbach's Alpha Coefficient for this scale was 0.853.

#### 2.2.2. Anxiety symptoms

The Chinese version of the Generalized Anxiety Scale (GAD-7) was used to investigate the anxiety symptoms of the study participants in the past 2 weeks. The scale includes seven items, with a total score ranging from 0 to 21. The severity of anxiety symptoms increases as the score rises. A score of ≥10 is the cut-off value for a positive anxiety symptom screening (58). Foreign research has confirmed the reliability and validity of the scale (59, 60). This scale has also been widely used in China and shows good reliability and validity (61, 62). In this study, Cronbach's Alpha Coefficient for this scale was 0.899.

#### 2.2.3. Suicidal ideation

A team of experts led by Harel-Fisch, O'Carroll, and Waxweiller developed the suicidal ideation assessment entries for the Adolescent Risk Behavior Surveillance System (63, 64). That is, during the past 12 months, did you attempt?

### 2.3. Interpersonal relations

#### 2.3.1. Parent-child relationships

Adapted from the National Longitudinal Study of Adolescent to Adult Health (Add Health), the parent-child relationship questionnaire examines the quality of adolescents' self-reported interactions with parents using 12 original items. In foreign studies, the α coefficient of Cronbach is 0.88 (65, 66). Five items were selected and adapted for this study, including the subjective entry “How satisfied are you with your relationship with your mother/father?” (1–10 points). Our team chose and modified five items in this study, including the subjective personal item “Mother/Father Relationship Satisfaction Score” (1–10 points). The relationship satisfaction scores of 1–3 were deemed unsatisfactory, while those of 9–10 were deemed ideal. The following three questions are “how frequently do you communicate with your mother/father” and “how cohesive are the family members.” The level of cohesion among family members can be categorized as low, medium, or high. The Cronbach α coefficient of the study's research project is 0.702.

#### 2.3.2. Peer relationships

We used the Global School Student Health Survey (GSHS), sponsored by the World Health Organization (WHO) and studied by the Centers for Disease Control and Prevention, to understand the relationships between middle school students and their classmates. It has been used by Chinese middle school students (67). We extracted two questions: “How many close friends do you have?” and “How do you view classmates to be caring?” We added two questions: “Rate your level of satisfaction with your relationship” and “How cohesive is your class?” In this study, Cronbach's Alpha Coefficient for this scale was 0.579.

#### 2.3.3. Teacher-student relationships

Two teacher-student relationship items adapted from those used in the American Adolescent Health Cohort (Add Health) were used to examine the self-reported quality of teacher-student relationships among adolescents. Cronbach α coefficient was 0.76, and it was applied to the middle school population in China (65–68). Our team adapted this item to include “the satisfaction score of your relationship with the head teacher” and “do you perceive caring by your school teacher?” In this study, Cronbach's Alpha Coefficient for this scale was 0.539.

### 2.4. Statistical analysis

Using frequency composition ratios to describe the socio-demographic characteristics of the study's participants. The Chi-square test is used to examine the correlation between interpersonal relationships and depression and anxiety symptoms, with relevant factors included in the subsequent analysis. In chi-square test analysis, principal component analysis (PCA) extracts principal components from meaningful interpersonal variables. SPSS 23.0 software performs the analysis as described previously. The path model of interpersonal relationships and depression and anxiety symptoms was built using AMOS 17.0 software, and the normality of all variables was tested before analysis. All statistical test levels are α= 0.05, and *p* ≤ 0.05 indicates a statistical difference.

## 3. Results

### 3.1. Characteristics of participants

In this study, 71 classes, including 3,669 middle school students, were recruited, and 3,480 completed the survey, with a response rate of 94.8%. Among the 189 students who didn't respond, 80 did not agree to participate in the survey, 65 failed to submit the questionnaire due to an unstable network, and 44 did not participate due to a leave of absence. One thousand seven hundred seven of the 3,480 students who completed the survey were female (49.1%), 2,396 were high school students (68.9%), and 1,707 had no brothers or sisters (54.8%). The fathers of 1,535 participants had a bachelor's degree or higher (44.1%), and the mothers of 306 participants had a bachelor's degree or above (37.5%). Seven hundred five participants had strong academic performance (20.3%), and 389 participants had bad academic performance (11.2%); 3,140 participants' parents' marital status was stable (90.2%). The sociodemographic characteristics of the samples are shown in Table 1.

**Table 1:** Socio-demographic characteristics of participants (*N* = 3,480).

**Characteristics**	**Group**	**Count (%)**
Gender	Males	1,773 (50.9)
	Females	1,707 (49.1)
Grade	Grade one	872 (25.1)
	Grade two	211 (6.1)
	Senior grade one	2,119 (60.9)
	Senior grade one Two	278 (8.0)
Age (years)	11–14	1,049 (30.1)
	15–16	1,948 (55.98)
	17–20	483 (13.9)
Do parents have only one child	Yes	1,907 (54.8)
	No	1,573 (45.2)
Dwelling state	Live with your parents	2,046 (58.8)
	Live with others	1,379 (39.6)
	live alone	55 (1.6)
Learning achievement	Good	705 (20.3)
	Medium,	(68.6)
	Poor	389 (11.2)
Marriage status of parents	Stabilize	3,140 (90.2)
	Instability	340 (9.8)
Father's degree	High school and below	1,945 (55.9)
	University degree or above	1,535 (44.1)
Mother's degree	High school and below	2,174 (62.5)
	University degree or above	1,306 (37.5)

### 3.2. Chi-square test results

#### 3.2.1. Interpersonal relations and depressive symptoms

The results of the chi-square test for interpersonal relationships and depressive symptoms are displayed in Table 2. Students with a low frequency of communication with their parents (less than once a week), low satisfaction with parental relationships, classmates, and teachers, and low cohesion among family members and classes, few close friends (0–2), and those who did not feel cared for by classmates and teachers had a higher prevalence of depressive symptoms.

**Table 2:** The association between interpersonal relations and depression and anxiety symptoms in middle school students (*N* = 3,480).

**Variable**	**Depressive symptoms** ***N* = 595 *n* (%)**	***P*-value**	**Anxiety symptom** ***N* = 499 *n* (%)**	***P*-value**	**Suicidal ideation *N* = 316 *n* (%)**	***P*-value**
**Satisfaction with relationship to mother**		**<0.001**		**<0.001**		**<0.001**
1–3 points	24 (35.8)		19 (28.4)		21 (31.3)	
4–8 points	285 (23.3)		226 (18.5)		154 (12.6)	
9–10 points	286 (13.1)		254 (11.6)		141 (6.4)	
**Satisfaction with relationship to father**		**<0.001**		**<0.001**		**<0.001**
1–3 points	58 (39.5)		42 (28.6)		38 (25.9)	
4–8 points	313 (21.0)		262 (17.6)		154 (10.3)	
9–10 points	224 (12.1)		195 (10.6)		124 (6.7)	
**Communication frequency with mother**		**<0.001**		**<0.001**		**<0.001**
Less than once a week	117 (26.5)		94 (21.3)		73 (16.6)	
Once a week	305 (18.1)		250 (14.8)		136 (8.1)	
Twice a week at least	173 (12.8)		155 (11.4)		107 (7.9)	
**Communication frequency with father**		**<0.001**		**<0.001**		**<0.001**
Less than once a week	235 (24.8)		193 (20.3)		129 (13.6)	
Once a week	272 (15.4)		218 (12.4)		135 (7.7)	
More than once a week	88 (11.5)		88 (11.5)		52 (6.8)	
**Cohesion among family members**		**<0.001**		**<0.001**		**<0.001**
Low	21 (30.0)		14 (20.0)		14 (20.0)	
Medium	190 (28.8)		134 (20.3)		106 (16.1)	
High	384 (14.0)		351 (12.8)		196 (7.1)	
**Cohesion among the class**		**<0.001**		**<0.001**		**<0.001**
Low	23 (32.4)		14 (19.7)		143 (7.6)	
Medium	334 (21.8)		268 (17.5)		160 (10.4)	
High	238 (12.7)		217 (11.6)		123 (10.0)	
**Number of close friends**		**<0.001**		**<0.004**		**0.038**
0–2	121 (24.7)		100 (20.4)		64 (13.1)	
3–6	258 (16.8)		229 (14.9)		140 (9.1)	
7 or more	60 (17.9)		43 (12.8)		32 (9.5)	
**Satisfaction with relationship to friends**		**<0.001**		0.081		0.516
1–3 Points	6 (22.2)		3 (11.1)		3 (11.1)	
4–8 Points	184 (20.6)		148 (16.6)		89 (10.0)	
9–10 Points	405 (15.8)		348 (13.6)		224 (8.8)	
**Perceived caring by classmates**		**<0.001**		**<0.001**		0.136
No	249 (22.6)		201 (18.2)		115 (10.4)	
Occasional	250 (14.9)		212 (12.7)		148 (8.8)	
Often	96 (13.6)		86 (12.2)		53 (7.5)	
**Satisfaction with relationship to head teacher**		**<0.001**		**<0.001**		**<0.001**
1–3 Points	69 (37.1)		49 (26.3)		33 (17.7)	
4–8 Points	416 (17.9)		359 (15.5)		220 (9.5)	
9–10 Points	110 (11.3)		91 (9.4)		63 (6.5)	
**Perceived caring by school teachers**		**<0.01**		**<0.01**		**<0.01**
No	32 (37.2)		24 (27.9)		18 (20.9)	
Occasional	37 (37.0)		25 (25.0)		15 (15.0)	
Often	48 (29.1)		34 (20.6)		23 (13.9)	

The bold values indicated to highlight the significant difference values of *p* < 0.05.

#### 3.2.2. Interpersonal relations and anxiety symptoms

The chi-square test of interpersonal relationships and anxiety symptoms revealed 10 statistically significant interpersonal variables. Among parent-child relationships, anxiety symptoms were found to be more prevalent in students with low relationship satisfaction and low communication frequency with their parents. In contrast, a high degree of cohesiveness within the family was associated with a low prevalence of anxiety symptoms. In peer relationships, anxiety symptoms are uncommon among students with low-class cohesion, few friends, and no sense of peer care. Among teacher-student relationships, anxiety symptoms were more prevalent among students who were dissatisfied with their head teacher and did not sense concern from their school teachers. Table 2 displays the specific results.

#### 3.2.3. Interpersonal relations and suicidal ideation

The chi-square test for interpersonal relationships and suicidal ideation revealed nine variables with statistically significant differences. In parent-child relationships, students who are dissatisfied with their relationship with their parents and seldom communicate with them exhibit more anxiety symptoms. In contrast, those close to family members have a lower incidence of anxiety symptoms. In peer interactions, anxiety symptoms are uncommon among students with low-class cohesion and few friends. Suicidal ideation was more prevalent among students who were dissatisfied with their teachers and did not feel cared for by their teachers. Table 2 shows the results in detail.

### 3.3. Path analysis results

On 11 significant variables, principal component analysis and dimension reduction were performed before the path analysis. There are four dimensions of interpersonal relationships: mother-child, father-child, companion, teachers, and students. For further analysis, the common factors of the four dimensions are replaced with the original variables. The greater the total load of the factor extraction, the more significant the contribution of this principal component and the greater the amount of information kept from the original data. In this paper, the sum of squares of extracted loads of the male factors selected for the four dimensions of the mother-child relationship, father-child relationship, peer relationship, and teacher-student relationship were 60.8, 60.3, 46.8, and 74.3%, respectively, representing the largest component of the sum of squares of extracted loads of this dimension. Supplementary Table 1 displays each dimension's detailed factor loading matrices.

Based on the literature analysis, we created an association model of interpersonal relationships with anxiety, depression, and suicidal ideation in all subjects and grades (junior high and senior high school). We verified the model with the software Amos 23.0. According to Supplementary Table 2, the fitting indexes of the three models are all ideal, which indicates that the models constructed could well describe the effects and influencing paths of the mother-child relationship, father-child relationship, peer relationship, and teacher-student relationship on anxiety symptoms, depression symptoms, and suicidal ideation.

**Figure 1:**
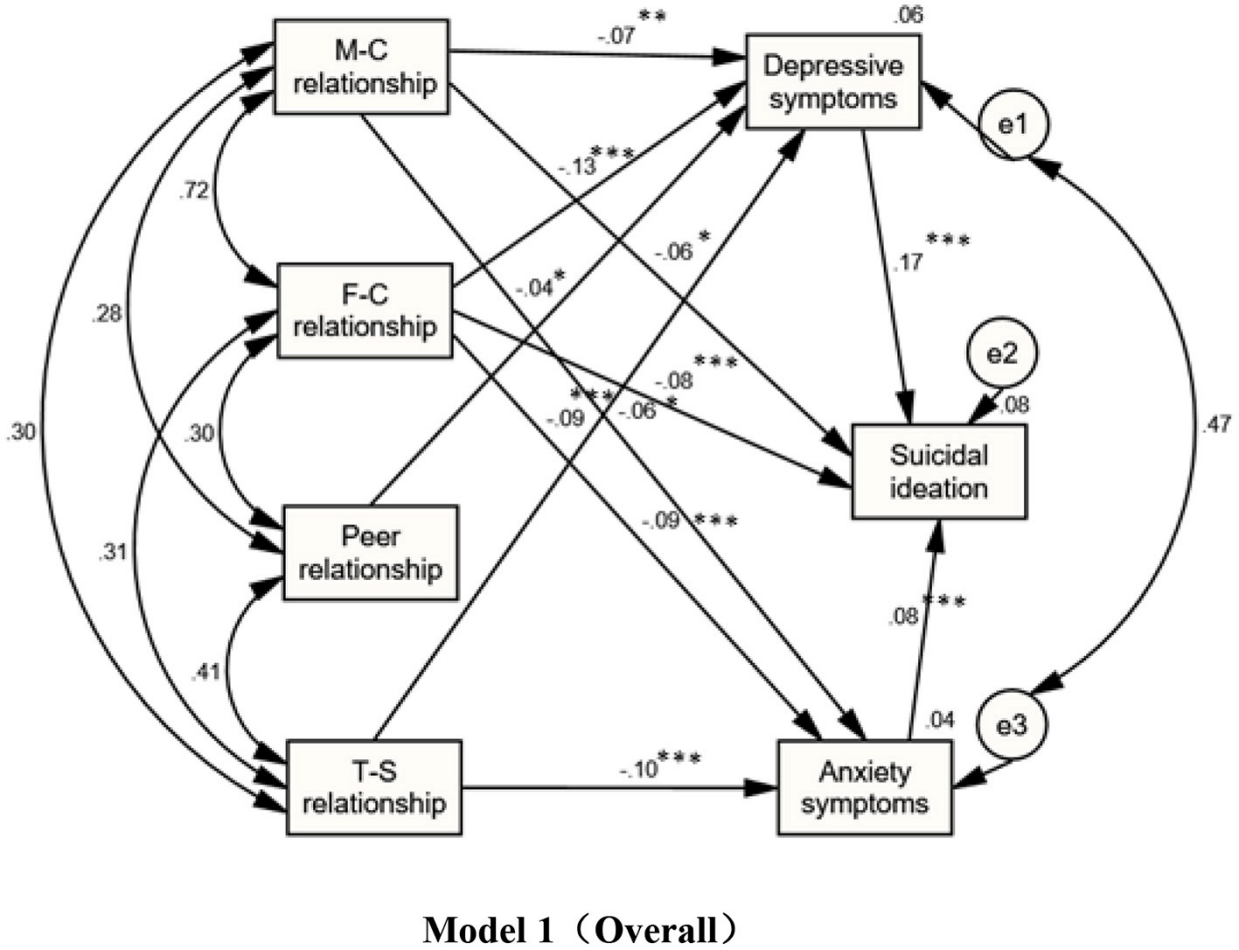
Structural equation model diagram and standardized path coefficient for correlation between interpersonal relations and depression, anxiety symptoms and suicide ideation (M-C = Mother-Child; F-C = Father-Child; T-S = Teacher-Student). *P < 0.05, **P < 0.01, ***P < 0.001; All coefficients are standardized coefficients.

**Figure 2:**
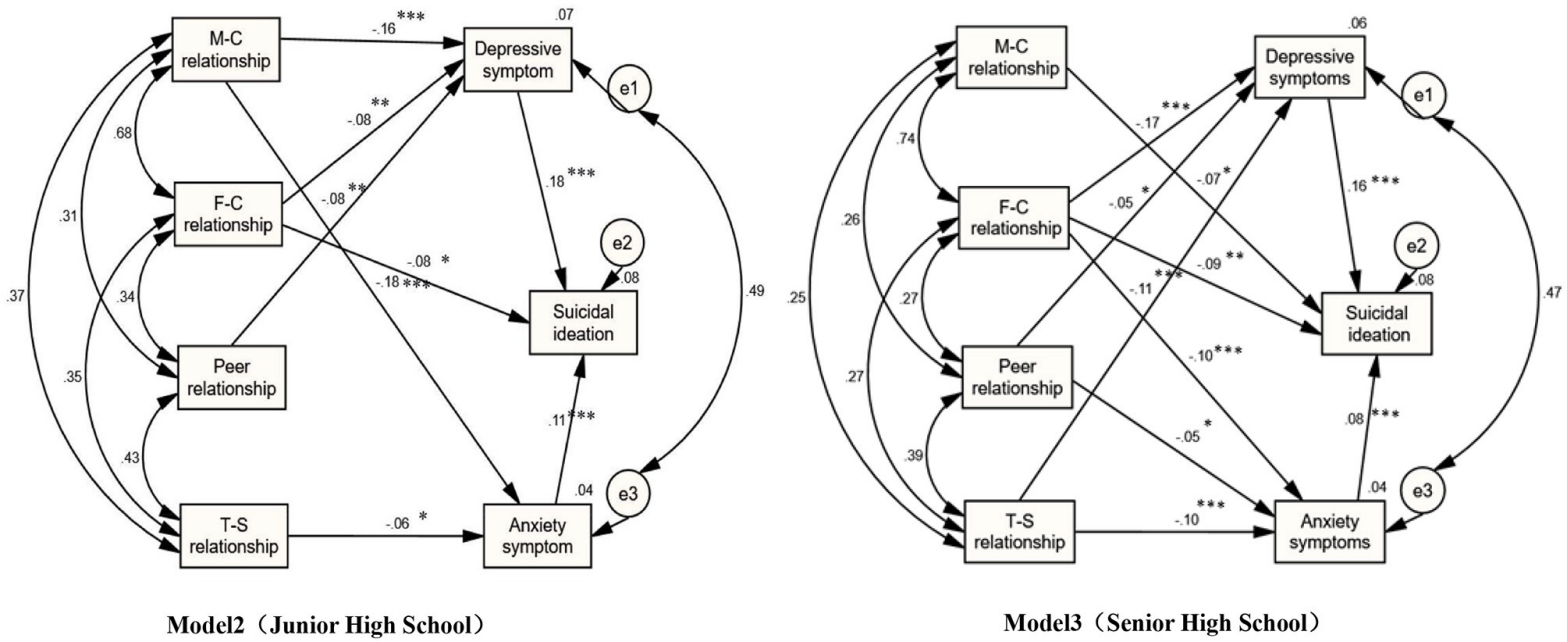
Path analysis method is used to analyze the interpersonal relationship, anxiety, depression and suicidal ideation of middle school students by grade. Model 2 was a structural equation model for junior high school students' interpersonal relationship and anxiety symptoms, depression symptoms, and suicidal ideation. Model 3 was a structural equation model of high school students' interpersonal relationship and anxiety symptoms, depression symptoms, and suicidal ideation (M-C = Mother-Child; F-C = Father-Child; T-S = Teacher-Student). *P < 0.05, **P < 0.01, ***P < 0.001; All coefficients are standardized coefficients.

Refer to Figures 1, 2 and Table 3 for the results of the model fitting correction path analysis of the interpersonal relationship, depression, and anxiety symptoms of middle school students. The standard path coefficients for the direct influence of the mother-child relationship on anxiety, depression, and suicidal ideation are −0.06, −0.07, and −0.06, according to the analysis presented in Table 3. In the structural model of path analysis, the standardized indirect effect value is equal to the multiplication of the path coefficient β value, and the indirect effect of the mother-child relationship on suicidal ideation through anxiety symptoms was −0.06 × 0.08 = −0.0048. The indirect impact of the mother-child relationship on suicidal ideation through depressive symptoms was – 0.07 × 0.17 = −0.0119, and the size of the indirect effect of the mother-child relationship on suicidal ideation was −0.016. For junior high school students, the standard path coefficients of the mother-child relationship directly affecting anxiety and depression are −0.182 and 0.156, respectively; The indirect effect of the mother-infant relationship on suicidal ideation through anxiety symptoms is −0.182 × 0.109 = 0.0198; The indirect effect of the mother-child relationship on suicidal ideation through depressive symptoms was −0.156 × 0.184 = −0.0287, and the indirect effect of the mother-child relationship on suicidal ideation was −0.049. The direct effect of the mother-child relationship on suicidal ideation among high school students was represented by a standard path coefficient of −0.073.

**Table 3:** Effect value of path analysis of interpersonal relations and emotional problem.

**Path**	**Model 1 (Overall)**			**Model 2 (Junior High School)**			**Model 3 (Senior High School)**		
	**TE**	**DE**	**IE**	**TE**	**DE**	**IE**	**TE**	**DE**	**IE**
M-C relationship → Anxiety symptoms	−0.056	−0.056		−0.182	−0.182				
M-C relationship → Depressive symptoms	−0.074	−0.074		−0.156	−0.156				
M-C relationship → Suicidal ideation	−0.076	−0.059	−0.017			−0.049	−0.073	−0.073	
F-C relationship → Anxiety symptoms	−0.086	−0.086					−0.104	−0.104	
F-C relationship → Depressive symptoms	−0.133	−0.133		−0.083	−0.083		−0.174	−0.174	
F-C relationship → Suicidal ideation	−0.109	−0.080	−0.03	−0.093	−0.078	−0.015	−0.123	−0.086	−0.036
Peer relationship → Anxiety symptoms							−0.055	−0.055	
Peer relationship → Depressive symptoms	−0.037	−0.037		−0.075	−0.075		−0.050	−0.050	
Peer relationship → Suicidal ideation	−0.006		−0.006	−0.014		−0.014	−0.012		−0.012
T-S relationship → Anxiety symptoms	−0.098	−0.098		−0.057	−0.057		−0.095	−0.095	
T-S relationship → Depressive symptoms	−0.087	−0.087					0.111	−0.111	
T-S relationship → Suicidal ideation	−0.023		−0.023	−0.006		−0.006	−0.026		−0.026
Anxiety symptoms → Suicidal ideation	0.085	0.085		0.109	0.109		0.077	0.077	
Depressive symptoms → Suicidal ideation	0.169	0.169		0.184	0.184		0.164	0.164	

TE, Total effect; DE, Direct effect; IE, Indirect effect; M-C, Mother-Child; F-C, Father-Child; T-S, Teacher-Student.

The standard path coefficients for the father-child relationship's direct effect on anxiety symptoms, depressive symptoms, and suicidal ideation were −0.09, −0.13, and −0.08, respectively. The indirect effect of the father-child relationship on suicidal ideation through anxiety symptoms was −0.09 × 0.08 = −0.0081, while the indirect effect of the father-child relationship on suicidal ideation through depressive symptoms was −0.13 × 0.17 = −0.0221. The indirect effect size of the father-son relationship on suicidal ideation was −0.03. The standard path coefficients of the father-child relationship's direct influence on depression symptoms and suicidal ideation in junior middle school students were −0.083 and −0.078, respectively. The father-child relationship affected suicidal ideation indirectly through depressive symptoms, with an indirect effect of −0.083 × 0.184 = 0.0153; The indirect effect of the father-child relationship on suicidal ideation was −0.015. For high school students, the standard path coefficients of the father-child relationship's direct influence on anxiety, depression, and suicidal ideation were −0.104, −0.174, and −0.086; The father-child relationship had an indirect effect on suicidal ideation through anxiety symptoms, and the indirect effect was 0.104 × 0.077 = 0.008; The father-child relationship indirectly affected suicidal ideation through depressive symptoms, and the indirect effect was −0.174 × 0.164 = −0.029. The father-son relationship had an indirect influence on suicidal ideation of 0.036%.

The standardized path coefficient for the direct influence of peer relationships on depressive symptoms was −0.037, and the standardized path coefficient for the indirect influence on suicidal ideation through depressive symptoms was −0.037 × 0.17 = −0.0063. The direct effect of peer relationships on anxiety symptoms and suicidal ideation was insignificant. For junior high school students, the standard path coefficient of the direct effect of peer relationships on depression symptoms was −0.075; Peer relationships had an indirect influence on suicidal ideation through depressive symptoms, and the indirect effect was 0.075 × 0.184 = 0.0138; The indirect effect of peer relationship on suicidal ideation was 0.014. The standard path coefficient of the direct influence of peer relationships on anxiety and depression was −0.055 and −0.050 among senior high school students. Peer relationships influenced suicidal ideation indirectly through anxiety symptoms, with an indirect effect of 0.05 × 0.07 = 0.004. The indirect effect of peer relationships on suicidal ideation was 0.05 × 0.164 = −0.008. The indirect effect of peer relationships on suicidal ideation was 0.012.

The standard path coefficients of the teacher-student relationship's direct influence on anxiety and depression symptoms are −0.98 and −0.87, respectively. However, the direct effect path of the teacher-student relationship on suicidal ideation was not significant. On the contrary, the indirect influence of the teacher-student relationship on suicidal ideation through anxiety and depression symptoms was −0.023. The standard path coefficient of the direct effect of the relationship between middle school students and teachers on anxiety symptoms is −0.057. Teacher-student relationship indirectly affected suicidal ideation through anxiety symptoms, with an indirect effect of −0.057 × 0.077 = 0.004. The indirect impact of the teacher-student relationship on suicidal ideation is 0.006. The standard path coefficients of the direct effect of the teacher-student relationship on anxiety and depression for senior high school students are −0.095 and −0.111, respectively. The indirect impact of the teacher-student relationship on suicide ideation through anxiety symptoms was −0.095 × 0.077 = 0.007, the indirect effect of the teacher-student relationship on suicide ideation through depression symptoms was −0.111 × 0.164 = 0.018, and the indirect effect of the teacher-student relationship on suicide ideation was −0.026.

The direct effect of anxiety and depression symptoms on suicidal ideation was represented by standard path coefficients of 0.085 and 0.164, respectively. The direct effects of anxiety and depression on suicidal ideation in junior high school were represented by standard path coefficients of 0.109 and 0.184, respectively. For the direct effect of anxiety and depression on suicidal ideation in high school, the standard path coefficients were 0.077 and 0.164, respectively.

## 4. Discussion

According to studies, interpersonal relationships are strongly associated with depressive, anxious, and suicidal ideation. The father-child relationship influences depression symptoms the most, followed by the mother-child relationship, the teacher-student relationship, and the peer relationship. The relationship between teachers and students significantly impacts students with anxiety symptoms, followed by the relationship between fathers and sons. In terms of suicidal ideation, the parent-child relationship has a direct negative impact on suicidal ideation; the Parent-child relationship, peer relationship and teacher-student relationship have an indirect negative influence on suicidal ideation through anxiety and depression symptoms; In the parent-child relationships, the father-son relationship has the most significant influence on suicidal ideation, followed by mother-child relationship.

In addition, the effect of interpersonal relationships on anxiety symptoms, depressive symptoms, and suicidal ideation varies dramatically by grade. The mother-child relationship had the most significant impact on anxiety symptoms among junior high school students, followed by the teacher-student relationship. Among depression symptoms, the mother-child relationship has the most crucial influence on depression symptoms, followed by the father-child relationship and peer relationship. Only the father-son relationship influences suicidal ideation significantly, whereas other interpersonal relationships have no significant effect on suicidal ideation. Father-son relationships have the greatest influence on anxiety and depression among senior high school students, followed by teacher-student and peer relationships. The father-child relationship significantly impacts senior high school students' suicidal ideation, followed by the mother-child relationship. This study's results are discussed as follows:

### 4.1. Interpersonal relations and depressive symptoms

Middle school students' interpersonal relationships are closely linked to depressive symptoms. Xiang et al. demonstrated that the teacher-student, peer, and parent-child relationships of students in secondary school were negatively related to depression symptoms (69). It is challenging to explain parent-child interactions and relationships with a single interpersonal variable. In the parent-child interaction process, the relationship between children and their parents and the frequency of communication influence teenagers' learning experiences and life (43). The present study revealed that the self-perceived degree of relationship with parents and objective frequency of chatting was significantly related to depressive symptoms. Poor parent-child relationships and infrequent chatting were risk factors for depressive symptoms. Poor parent-child relationships and friendship quality were risk factors for depression in adolescents with depression, according to research by Xiuyue et al. (30). During the development of self-identity, peer groups provide emotional support and identity. The theory of socialization development emphasizes the significance of peer relationships in the interpersonal communication of middle school students (70). This study revealed that low-class closeness, few friends, low relationship scores with the closest friends, and poor peer relationships increased the risk of depressive symptoms (70). Although studies have demonstrated that the level of interaction between middle school students and teachers is always low (49), teacher-student relationships are significant in adolescents because teachers are the focal point of students' learning paths and monitor their academic performance. Students have more contact with their teachers at school than with their parents and friends. Students who had a poor teacher-student relationship with their classroom teachers and those who had a poor relationship with their school teachers had a higher prevalence of depressive symptoms, according to the results of this study.

Considering that interpersonal relationships may affect the depressive symptoms of students in different grade levels. Therefore, we also conduct further hierarchical analysis according to grade. The mother-child relationship had the most significant impact on depressive symptoms among junior middle school students, followed by the father-son relationship and the peer relationship. It is consistent with our expectations and previous research findings. Junior high school students spend more time with their parents than seniors, and mothers have a stronger interaction and emotional connection with teenagers than fathers. Therefore, the relationship between mother and child is typically closer than that between father and son. However, at the same time, the mother is more likely to have more conflicts and interactions with junior high school students (49). It was found that mother-child tension was one of the most significant risk factors for depression among middle school students (71, 72). Therefore, their mother plays a more critical role in their education in junior high school than their fathers. The father-son relationship significantly influenced depressive symptoms throughout senior year of high school, followed by the teacher-student relationship and the peer relationship. Laursen et al. (72) found that most high school students are bound by the values of examination-first parents and teachers and live a reading-centered life. High school students at this stage are more likely to suffer from depression than junior high students (72). Furthermore, this study indicated that the mother-child relationship had no direct effect on the depressive symptoms of senior high school students. This may be because mothers often spent more time with teenagers, participated in more senior high school students' recreational activities, and provided more care and interactive support than fathers. In the parent-child interaction process, the degree of relationship between children and their parents and the frequency of interaction can influence teenagers' learning and life experiences, according to Laursen et al. (72). In comparison to mothers, fathers spent less time with senior high school students but provided greater social support in the form of tools or learning opportunities (72). Since high school students' rest time and extracurricular activities are occupied by studies, the influence of the father-son relationship on depressive symptoms of high school students is more significant.

Therefore, given the various developmental stages of middle school students, mental health education requires the assistance of schools, teachers, and parents. School mental health workers should focus on students' interpersonal relationships, particularly parent-child relationships while paying attention to students' current mental health status.

### 4.2. Interpersonal relations and anxiety symptoms

Symptoms of anxiety are also common among adolescence. According to the results of this research, the self-reported parent-child relationships with mothers and fathers and the frequency of talking with mothers and fathers are related to anxiety symptoms. Li et al. demonstrate that the relationship between parent-child relationships and anxiety symptoms varies between urban and rural areas. Urban students' parent-child relationships are associated with anxiety, whereas rural students' parent-child relationships are associated with anxiety (73). The degree of intimacy with the class, the number of friends, and the relationship with classmates are associated with anxiety symptoms in peer relationships. The research by Yang et al. also demonstrated that teacher-student and peer relationships are closely related to the anxiety of middle school students. Good peer relationships alleviate anxiety symptoms in students (73). Peer relationships are similar to parent-student and teacher-student relationships because peers can provide excellent emotional support, companionship, and encouragement. Peers do not necessarily assist high school students in addressing and dealing with sources of anxiety, although confiding in peers helps alleviate anxiety symptoms.

This study conducted a path analysis of the association between interpersonal relationships and anxiety symptoms among students of varying grade levels. Consistent with Li et al., mother-child relationships among junior high school students had the most significant impact on anxiety symptoms, followed by teacher-student relationships (73). The parent-child relationship had a significant effect on the anxiety symptoms of junior high school students but had no significant impact on anxiety symptoms. At the junior middle school stage, the mother and father's parenting styles and the frequency and content of their communication with their children differ, causing them to provide their children with different emotional support and assistance in their studies and daily lives. Therefore, the mother-child and father-child relationships have distinct effects on students' anxiety. When teenagers are young, moms pay more attention to their children than fathers, according to research by Liu et al. Although this situation will result in teenagers getting significantly more support from their mothers than their fathers, it will also result in more mother-child conflicts than father-child conflicts at this stage (74). Unlike depressive symptoms, teacher-student relationships significantly impacted junior high students' anxiety symptoms. Self-perception was associated with a poor relationship and anxiety symptoms between the head teacher and the school teacher. A poor relationship between the head teacher and the school teacher was a risk factor for anxiety symptoms in middle school students. Yang et al. found that the teacher-student relationship had a significant impact on the anxiety of junior high school students, with the negative emotion in the teacher-student relationship having the most significant impact, followed by the positive emotion (75). For us, enlightenment alleviates the anxiety symptoms of junior high school students. We should also focus on cultivating the emotions of teachers and students and improving the relationship between teachers and students.

The father-child relationship had the greatest influence on the anxiety symptoms of senior high school students, followed by the teacher-student relationship and the peer relationship. According to the 2009 Comparative Study on the Rights and Interests of Senior High School Students in China, Japan, South Korea, and the United States released by the China Youth Research Center, among the top five high school students from all over the world, Japan, South Korea and the United States ranked their father as the fifth, while high school students from China preferred confiding their troubles to netizens, with their father ranking the sixth (Research Group of China Youth Research Center, 2009). Fathers and Chinese college students are estranged. The traditional feudal concept may impact the father-son relationship in China. Specifically, some fathers continue to stick to the “strict father's loving mother” principle and always discipline their children with rigorous discipline, even acting as the family's rulers, which is simplistic and impolite and alienates the father-son relationship (76). Therefore, many fathers and sons cannot have a healthy relationship, which is detrimental to children's growth. Melanie Keller, a professor of psychology at California State University, published a study at the annual meeting of the American Psychological Association demonstrating that fathers have a significant impact on children's mental health: a good father-son relationship makes children more resilient to adversity as they mature. This study showed that the self-perceived relationship with the father and family and the objective frequency of chatting are strongly associated with depressive symptoms. The more distant the self-perceived relationship, the less frequently individuals chat, which is a risk factor for depression. Therefore, the estrangement between father and son may significantly affect middle school students' anxiety and depression symptoms. In this study, peer relationships have no effect on anxiety symptoms in junior high school students, while their contribution to anxiety symptoms in high school students is small. Although Chi-square analysis of peer relationship and anxiety symptoms indicates that low intimacy with class, few friends, low scores with good friends, and poor relationships with classmates increase the risk of depression symptoms, the path analysis of peer relationship and anxiety symptoms demonstrates that peer relationship has a very weak influence on middle school students' future emotional problems comp (77, 78).

In addition, related research shows that in the teacher-student relationship, the relationship between self-perception and classroom and school teachers is related to anxiety symptoms, and a poor relationship between the classroom and school teachers is a risk factor for the anxiety of middle school students. In this study, the impact of the teacher-student relationship on anxiety symptoms was considerable across middle school students' developmental stages (junior high school and senior high school). This could be because the instructor and supervisor of the middle school students' studies do not care much about the students. Students with good grades and fast progress get more attention and encouragement from teachers. Middle school students' primary reason for anxiety is their academic achievements, which influence their views on teacher-student relationships. Thus, students' perceptions of teacher-student relationships are more closely associated with anxiety symptoms (75). The significant correlation between the teacher-student relationship and middle school students' anxiety symptoms demonstrates the necessity of focusing on the teacher-student relationship in mental health work with children. Work in mental health is not only the responsibility of psychology teachers but also of classroom teachers.

### 4.3. Association between interpersonal relations and suicidal ideation

The association analysis of the mother-child relationship, father-child relationship, peer relationship, and suicidal ideation of all the students revealed that the father-son relationship had the greatest influence on suicidal ideation, followed by the mother-child relationship and peer relationship. In contrast, the teacher-student relationship had no significant influence on suicidal ideation. The results of a longitudinal study on adolescent health in the United States indicate that the parent-child relationship was an important protective factor for middle school students' suicide attempts, whereas the relationships between peers and teachers and students have a more moderating effect (41). Using hierarchical linear modeling, Israeli scholar Madjar analyzed the impacts of three interpersonal relations on suicidal ideation and Behavior in a school context, demonstrating that parental and peer support were associated with students' suicidal ideation at the individual level (42). Poor parent-child relationships were more significantly associated with suicidal ideation and suicide attempts among Canadian adolescents aged 12–13 than poor peer relationships (79). Lang et al. identified parent/mother-child trust and communication as protective variables against suicidal ideation, but parent/mother-child estrangement was a risk factor. The critical role of parent-child relationships in adolescent suicidal ideation may be observed even though these studies used different measurement instruments and studied different populations, which precludes direct comparisons of results across countries.

Both fathers and mothers have essential roles in the development of middle school students, and father-child and mother-child relationships each play a unique role in the growth of their children. Father-child interaction is a vital component of parent-child activities and directly affects the quality of parent-child relationships (80). This study showed that parent-child relationships directly affect suicidal ideation. The higher the prevalence of suicidal ideation among students, the poorer the self-perceived relationship with parents and the less frequent the chats. Poor parent-child relationships may result in emotional troubles and affect depression and anxiety symptoms unrelated to the parent-child relationship. The sense of security of middle school students is largely dependent on the close relationship between family members, which can provide them with love and warmth when they are lonely and helpless and prevent them from developing suicidal ideation in response to negative emotions, thereby reducing the occurrence of suicidal ideation. In contrast, middle school students with poor parent-child relationships may exhibit less help-seeking behavior, poor treatment compliance, and a poor prognosis for therapy when they experience difficulties.

In addition, we studied each level according to grade and found that the father-child relationship had a stronger impact on the suicidal ideation of junior high school students. Father-child relationships contributed the most to suicidal ideation in high school, followed by mother-child relationships. According to a foreign study, as a child progresses through elementary school, junior high school, and senior high school, the focus of the educated in the family gradually shifts from the mother to the parents, considerably enhancing the father's effect on family education. Father is indispensable for children. In addition to being a nurturer, he is also a disciplinarian, a socialization, a mentor, a professional role model, and a consultant or partner in his spare time (81). In the world youth awareness survey, foreign youth hoped their father would value “family life” and work, whereas they hoped their mother would just value “family life.” Visible, being a model father is considerably more difficult than being a model mother (81). It may also explain why most students' relationships with their fathers are significantly less close than those with their mothers. In addition, Li et al. found (82) that the influence of father-child relationships on children's emotional and suicidal behavior problems increases after junior high school. Since the children began junior high school, the relationship between father and son has undergone a significant transformation. With the development of children's sociality, the increase of their cognitive level, and the expansion of their psychological needs, their needs and concerns have shifted from eating, drinking, having fun, and learning this information to social issues and their life development. Therefore, mothers can no longer provide for their children's needs, and the children wish to obtain them from their parents. This “turn” of junior high school students significantly increased the father's impact on the family's education. In the eyes of junior middle school students, the father is a nurturer and a model of socialization. Suppose the father-child relationship is good. In this case, the frequency of communication between children and their father increases trust between father and son, which can reduce the chance of children having suicidal ideation (83).

Moreover, this study demonstrated that interpersonal relationships indirectly affect suicidal ideation through anxiety and depression symptoms. Previous studies have shown that anxiety and depression are intermediate variables between peer relationships, parent-child relationships, and suicidal ideation (38). Regarding the mental health development of senior high school students, the importance of familial closeness cannot be overstated. Middle school students' mental health depends not only on school psychologists' guidance but also on the attention of society, schools, and families.

## 5. Limitations

Our study is the first comprehensive study to investigate the association paths and influence between different types of interpersonal relationships and middle school students' symptoms of depression, anxiety, and suicidal ideation. This study investigates the impact of interpersonal differences among middle school students at various developmental stages. Nonetheless, our study includes the following limitations:

Firstly, this study's samples only include students from four senior high schools, and their representativeness is limited. Secondly, this research data was collected between 2016 and 2017 and is old. With the development of time and the influence of the COVID-19 epidemic, the association between middle school students' interpersonal relationships and mental health issues may change. In addition, the middle schools included in this study are all urban, making it difficult to extend the results to rural areas or all middle schools. Therefore, future research can expand the sample size to include middle schools from rural areas and further explore and analyze the differences between urban and rural areas in interpersonal relationships, depression, and anxiety symptoms.

## 6. Conclusions

The father-child relationship affects suicidal ideation and depression the most, followed by the mother-child relationship, the teacher-student interaction, and the peer relationship. The teacher-student relationship influences anxiety symptoms the most, followed by the father-child and mother-child relationships. The association between interpersonal interactions and anxiety, depressive symptoms, and suicidal ideation varied significantly across grade levels.

## Data availability statement

The raw data supporting the conclusions of this article will be made available by the authors, without undue reservation.

## Ethics statement

The studies involving human participants were reviewed and the IRB approved the Xiangya School of Public Health study at Central South University. Written informed consent to participate in this study was provided by the participants' legal guardian/next of kin. Written informed consent was obtained from the minor(s)' legal guardian/next of kin for the publication of any potentially identifiable images or data included in this article.

## Author contributions

MZ, XG, ZC, and MH conceived and designed the study. MZ and XG analyzed the data. MH and JD provided guidance and support on data analysis. All authors were engaged in data collection, writing, and revising the study. All authors contributed to the article and approved the submitted version.
