# Supplementary material for: Association between interpersonal relations and anxiety, depression symptoms, and suicidal ideation among middle school students
Source: Front Public Health. 2023 Feb 14;11:1053341. doi: 10.3389/fpubh.2023.1053341 (PMC9971595; doi:10.3389/fpubh.2023.1053341)
Supplement: Supplementary file 1 [file Table_1.pdf]

Table 1 Interpersonal relations factor load matrix load matrix

Sum of squares / Variable	M-C relationship factor	F-C relationship factor	Peer relationship factor	T-S relationship factor
	60.78%	60.31%	46.8%	74.27%
Satisfaction with relationship to mother	0.827			
Frequency of communication with mother	0.742			
Closeness to family members	0.768	0.706		
Satisfaction with relationship to father		0.838		
Communication frequency with father		0.779		
Closeness to the class			0.742	
Number of close friends			0.621	
Satisfaction with relationship to friends			0.597	

Perceived caring by classmates	0.761	
Satisfaction with relationship to head teacher		0.862
Perceived caring by school teachers		0.862

Table 2 Model fitting index calculation results

**Fitting index of each model**	**$\chi 2$/df**	**NFI**	**IFI**	**TLI**	**CFI**	**GFI**	**AGFI**	**RMSEA**
Model 1（Overall）	2.267	0.999	0.999	0.995	0.999	0.999	0.995	0.019
Model 2（Junior High School）	5.090	0.997	1.001	1.002	1.000	0.999	0.994	0.000
Model 3（Senior High School）	6.287	0.998	0.999	0.997	0.999	0.999	0.995	0.015

*Note:*The overall model fit hypothesis test Bollen - Stine P-value was equal to 0.109, which was greater than 0.05.

χ^2/df= Chi-square/df; NFI= Normed fit index; IFI= Incremental Fit Index; TLI=Tucker-Lewis Index; CFI= Comparative Fit Index; GFI= The goodness-of-fit index; AGFI=Adjusted goodness-of-fit index; RMSEA= Root Mean Square Error of Approximation
